# Uremic leontiasis ossea due to secondary hyperparathyroidism complicated by vitamin C deficiency in a non-adherent chronic hemodialysis patient: A case report 

**DOI:** 10.5414/CNCS109788

**Published:** 2019-09-09

**Authors:** David Massicotte-Azarniouch, Laurie McLean, Pierre Antoine Brown

**Affiliations:** 1 Department of Medicine,; 2 Division of Nephrology, and; 3 Department of Otolaryngology – Head and Neck Surgery, University of Ottawa, Ottawa, ON, Canada

**Keywords:** hyperparathyroidism, vitamin C deficiency, leontiasis ossea, renal osteodystrophy, non-adherence, end-stage kidney disease

## Abstract

Non-adherence to medical therapy in patients with end-stage kidney disease (ESKD) can lead to severe metabolic derangements rarely seen in the current medical era. Such complications may take the form of secondary hyperparathyroidism (HPT) leading to rare manifestations of bone mineral disease, and profound vitamin C deficiency from poor nutrition combined with removal of water-soluble vitamins during dialysis. Secondary HPT causes renal osteodystrophy which can lead to diffuse enlargement of the facial skeleton and morphological changes suggestive of leontiasis ossea. We report a 36-year-old, non-adherent woman on chronic dialysis for over 10 years who developed progressive, diffuse facial bone enlargement in the context of years of extreme HPT and newly diagnosed severe vitamin C deficiency. Imaging revealed diffuse hypertrophy of the maxillary and mandibular bones. Histopathology showed extensive fibro-osseous proliferation without evidence of Brown tumor, suggestive of uremic leontiasis ossea. In this report, we discuss the orofacial manifestations of secondary HPT and the possible potentiating role of vitamin C deficiency on the development of renal osteodystrophy through altered vitamin D metabolism. Non-adherent patients on chronic dialysis should be evaluated for vitamin C deficiency, and the development of uremic leontiasis ossea should be considered when such patients present with distortion of facial features in the context of severe secondary HPT.

## Case report 

A 36-year-old Caucasian woman with end-stage kidney disease (ESKD) for 11 years was noted to have progressive enlargement of the lower face. She was known for primary focal segmental glomerulosclerosis (FSGS), diagnosed and treated at another center in another province, since the age of 3. Treatments had included courses of prednisone during early childhood and then cyclosporine from her teenage years to the age of 25. She was eventually started on hemodialysis (HD) via a central venous catheter (CVC) in 2007, then switched to peritoneal dialysis (PD) a year later. In 2013, after 5 years on PD, she had technique failure due to chronic non-adherence and a non-resolving catheter exit site infection. She re-initiated HD and shortly after, developed encapsulating peritoneal sclerosis for which she refused treatment with oral steroids and tamoxifen. She continued on HD via a CVC until she underwent creation of a left brachio-basilic arteriovenous (AV) fistula a year after re-initiation of HD. She continues to receive standard HD with a Fresenius FX800 filter via her fistula thrice weekly for 3.5 hours, with good adherence to her dialysis sessions. She has declined to consider renal transplantation. Other past medical history is significant for uremic encephalopathy and uremic pericarditis due to dialysis non-adherence, gastro-esophageal reflux disease, hypertension, ectopic pregnancy requiring unilateral salpingo-oopherectomy, migraines, as well as chronic non-adherence with renal diet and medications. 

**Figure 1.**
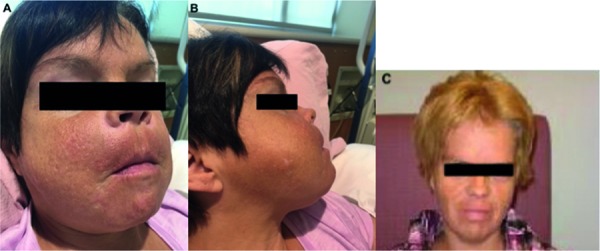
Facial morphology changes. Panels A and B are current photographs, whereas panel C is from 10 years earlier at start of dialysis.

**Figure 2.**
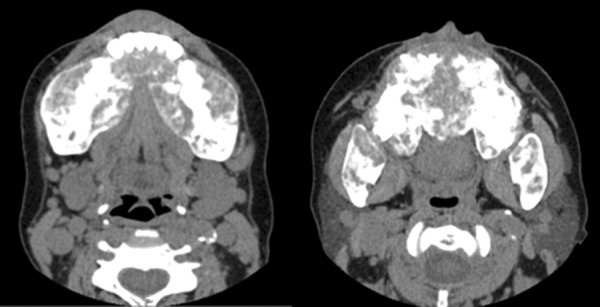
CT scan of the facial bones. The facial bones were enlarged, heterogeneous, sclerotic, and with some lytic areas, as well as bony expansion and tumefaction affecting most prominently the mandibular (left image) and maxillary areas, extending into the hard palate (right image).

Upon resumption of HD following PD failure, she had a progressively increasing parathyroid hormone (PTH) level, along with hyperphosphatemia, hypocalcemia, and elevated alkaline phosphatase (ALP) with liver function tests otherwise normal (Table 1). During this time, she was non-adherent to phosphate binders, calcimimetics (oral cinacalcet), and vitamin D analogues due to perceived side effects. The clinical picture was compatible with renal osteodystrophy. She declined imaging of the parathyroid and consideration of parathyroidectomy. In 2017, 4 years after resumption of HD, she noted jaw enlargement, tenderness and a change in facial features (Figure 1). She was evaluated by an internist who found gingival hypertrophy, an undetectable vitamin C level, and a low vitamin B12 level. There were no other signs of vitamin C deficiency such as gingival bleeding, diffuse folliculitis, or ecchymoses. She saw a maxillo-facial surgeon who proceeded to a partial resection of her maxillary and mandibular bone enlargements. Pathology revealed fibro-osseous, osteoblastic, and giant cell proliferation without evidence of Brown tumor. The clinical picture was suggestive of uremic leontiasis ossea. She initially felt improvement in her jaw symptoms, but had recurrence of these within 6 months of the surgery. A CT scan of the head and sinuses done at the time of symptom recurrence showed abnormality of all the bony structures (Figure 2). These changes were not present on a scan of the head and neck done 3 years prior. She was referred to an otorhinolaryngologist who found diffuse gingivitis (she had been off cyclosporine for over 10 years) and significant hypertrophy of the mandible, maxilla, and hard palate. It was recommended the patient undergo parathyroidectomy, but she adamantly refused. Repeat bone imaging of the head and neck a few months later showed worsening bony hypertrophy. The patient has been prescribed appropriate vitamin supplements including monthly intramuscular vitamin B12 in dialysis and 500 mg daily of oral vitamin C. While initially agreeable, she discontinued therapy after a few months. Her vitamin C levels remain undetectable at the time of writing this manuscript. She refuses longer or more frequent dialysis but accepts high dose IV calcitriol post-dialysis and claims to be adherent with oral vitamin D analogues and cinacalcet, which has led to some improvement in her biochemical markers (Table 1). She continues to categorically refuse imaging of or consideration for parathyroid surgery despite explanations and counselling from multiple physicians. She also declined psychological or psychiatric referral, denying there is any problem to be addressed. 

## Discussion 

**Figure 3.**
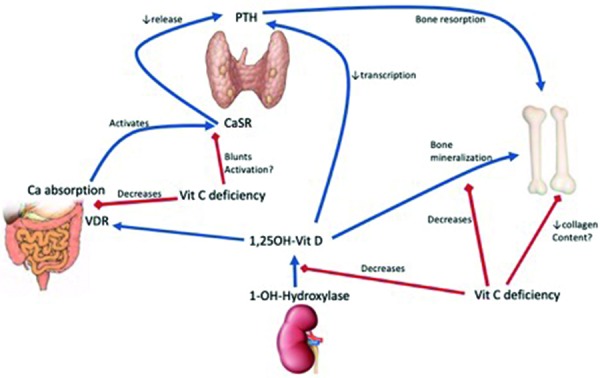
Role of vitamin C deficiency in vitamin D and PTH regulation. Vitamin C deficiency decreases 1-α-hydroxylase activity leading to decreased active vitamin D, which will in turn lead to greater transcription of PTH and less bone mineralization. Vitamin C deficiency also decreases the amount of vitamin D receptors (VDR) in the intestinal mucosal cells and the ability of active vitamin D to bind to the VDR in the intestine. This leads to a decrease in calcium absorption leading to hypocalcemia. Vitamin C deficiency may also blunt the activation of the calcium-sensing receptor (CaSR) by calcium, which could lead to higher levels of PTH. Finally, vitamin C deficiency may also lead to a decrease in collagen content of the bones through a decrease in hydroxyproline, a major constituent of bone collagen. Original figure produced by the study author DMA.

This case is a humbling reminder of the devastating consequences of uncontrolled hyperparathyroidism (HPT) and of the risks for modern-day scurvy in dialysis patients. Vitamin C (ascorbic acid) is a water-soluble vitamin removed through HD [1]. This, combined with poor nutrition and the fact that most vitamin C containing foods also contain potassium, leaves dialysis patients at high risk for vitamin C deficiency [2, 3]. In patients with ESKD, vitamin C deficiency may also lead to refractory anemia or erythropoietin resistance, possibly through decreased iron absorption and inflammatory oxidative stress [4, 5, 6]. Dialysis patients are frequently prescribed vitamin B and C complex supplements, and such supplementation has been associated with a 16% lower risk of mortality [7]. Vitamin C deficiency may also have potentiating effects on mineral bone disease associated with ESKD through alteration of vitamin D metabolism. Animal studies have demonstrated that vitamin C deficiency leads to a decrease in 1-α-hydroxylase activity in the kidney, a decrease in vitamin D receptors in the intestine, a decreased ability for active vitamin D to bind to these receptors, and the development of hypocalcemia [8]. It also leads to a decrease in bone mineralization and hydroxyproline content, a major component of bone collagen [8]. Furthermore, these effects were shown to persist despite adequate supplementation with vitamin D. Vitamin C deficiency has also been associated with the development of HPT in dialysis patients [3]. While this may be due to blunting of the effect of vitamin D on the intestine, it is proposed that vitamin C could also be involved in calcium-sensing receptor (CaSR) c-AMP-mediated signaling within the parathyroid gland. Deficiency may lead to a blunted response from the CaSR, inappropriately high PTH levels, and promote the development of renal osteodystrophy (Figure 3) [3]. Severe, prolonged secondary HPT leads to devastating musculoskeletal disease, including Brown tumors (localized osteolytic masses due to overactivation of osteoclasts). While the orofacial skeleton is not thought to be a site of predilection for renal osteodystrophy, these bones are not spared its ravages. Localized jaw pain and bone enlargement related to secondary HPT have been reported, with Brown tumors being the most common histopathological finding [9]. However, the soft tissue changes of the face related to chronic uremia may take on a more expansive, diffuse facial enlargement leading to the morphological changes described as uremic leontiasis ossea due to the resemblance to a lion’s face. The Sagliker syndrome, which likely represents another way of naming the same entity, has also been described [10]. We believe our case fits well the clinical, radiological, and pathological presentation of uremic leontiasis ossea as a consequence of years of severe, uncontrolled HPT. Leontiasis ossea is a clinical entity characterized by an enlargement of the mandibular and maxillary bones with flattening of the nasal ridge, giving a leonine facies. It may be present in the context of Paget’s disease, acromegaly, fibrous dysplasia or secondary HPT from ESKD (uremic leontiasis ossea). Uremic leontiasis ossea is a rarely reported manifestation of renal osteodystrophy [11, 12, 13, 14, 15, 16, 17, 18]. Histologically, it resembles fibrous dysplasia except for the presence of giant cells which suggest a reactive condition [19]. Radiographically, there is diffuse hypertrophy of the jaw bones, with ground-glass appearance and without the localized lytic masses typical of Brown tumors [20]. The bone enlargement can be severe enough to cause compressive neuropathies and even airway compromise [14, 15]. Treatment with parathyroidectomy only rarely leads to improvement in bone deformities [21, 22]. Most often it will at least offer stabilization of the condition [14, 23, 24, 25]. We hypothesize here that the concurrent presence of severe vitamin C deficiency in our patient was a potentiating factor for the development of such severe HPT and renal osteodystrophy. To our knowledge, such an association has never been reported. Given its proposed role in regulating vitamin D metabolism and PTH levels [3, 8], it is conceivable that a lack of vitamin C could potentiate renal osteodystrophy. Unfortunately, there is a paucity of data looking at the use of vitamin C in dialysis patients with HPT. One prospective observational study showed a significant reduction in PTH with the administration of vitamin C during dialysis [26]. A randomized, controlled trial of vitamin C given for 8 weeks vs. control in dialysis patients with HPT found no significant difference in PTH levels at 8 weeks [27]. However, the study has significant limitations related to a lack of methodological reporting making its results difficult to interpret. The potential association between vitamin C deficiency, altered vitamin D metabolism, and renal osteodystrophy requires further study. Our case highlights the devastating consequences of non-adherence to medical therapy in ESKD, the challenges in managing these patients and the importance of evaluating non-adherent patients for vitamin C deficiency. We believe it also highlights the potential benefits from expanding the role of psychological support for dialysis patients to ensure they are able to openly discuss concerns regarding their therapy which may affect adherence to routine medical recommendations. 

In conclusion, progressive facial morphology changes in dialysis patients should prompt consideration for the effects of renal osteodystrophy. This may manifest as diffuse enlargement of facial bones due to uremic leontiasis ossea. Furthermore, vitamin C deficiency is probably an underdiagnosed problem in dialysis patients. Vitamin C may play a role in the regulation of PTH and vitamin D metabolism, and severe deficiency may potentiate the development of renal osteodystrophy. 

## Consent 

Informed consent for the case report and pictures was obtained from the patient. 

## Acknowledgment 

We would like to thank Dianne Silverson, the dialysis team leader of the HD unit where the patient dialyzes, for obtaining the pictures and histopathologic reports relevant to this case, and all her excellent care for the patient. 

## Funding 

None. 

## Conflict of interest 

The authors declare that they have no relevant financial interests. 

**Table 1.** Chronology of events and biochemical markers.

	Total calcium (mmol/L)	Phosphate (mmol/L)	PTH (pmol/L)	ALP (U/L)	URR (%)	Vitamin C (µmol/L)
2007 Start of dialysis	2.03	3.16	58	78	–	
2010	2.09	2.27	117	101	–	
2013 PD failure, HD start	1.99	2.44	107	342	71	
2014	2.05	2.11	194	245	77	
2015	2.05	2.11	270	636	80	
2016	2.24	2.17	401	1,185	72	
2017	1.98	2.43	537	1,689	82	
2018	1.96	1.32	401	1,428	81	< 5 on 3 occasions

PTH = parathyroid hormone; ALP = alkaline phosphatase; URR = urea reduction rate.
